# Precise Manipulation of Bubble Cavitation and Blood–Brain Barrier Modulation using Temporal Interference Ultrasound

**DOI:** 10.34133/bmef.0323

**Published:** 2026-10-14

**Authors:** Jie Jin, Zhenxiang Ji, Zhetao Shangguan, Tianyi Yan, Shengyou Qian, Dingjie Suo

**Affiliations:** ^1^ School of Medical Science and Engineering, Beijing Institute of Technology, Beijing 100081, China.; ^2^ School of Physics and Electronics, Hunan Normal University, Changsha 410081, China.

## Abstract

**Objective:** To determine whether temporal interference (TI) ultrasound can enhance microbubble (MB) cavitation and provide transient modulation of blood–brain barrier (BBB) permeability. **Impact Statement:** By integrating a time-dependent multiphysics MB–vessel model with in vivo validation, this study links TI-enhanced cavitation dynamics to vessel-wall mechanical responses and a transient BBB permeability window, providing a mechanistic basis for pressure-efficient ultrasound-mediated brain delivery. **Introduction:** Focused ultrasound combined with MBs can transiently increase BBB permeability, but reliable control of cavitation intensity and post-sonication barrier recovery remains a key challenge for safe and effective brain delivery. **Methods:** A fluid–solid coupled MB–vessel model was used to evaluate MB dynamics, vessel-wall normal and shear stresses, porosity, acoustic-pressure dependence, and the influence of non-Newtonian blood rheology under 1.0 MHz single-frequency and 1.0 + 0.8 MHz TI excitation. In vivo Evans blue experiments in mice were then used to compare BBB opening across pressure levels and to characterize the recovery profile from 0.5 to 12 h after 0.3-MPa TI sonication. **Results:** Ultrasound-driven MB oscillation and collapse increased vessel-wall stress and modeled porosity. Compared with single-frequency ultrasound, TI excitation promoted earlier MB instability and collapse and produced stronger vessel-wall mechanical responses under energy-equivalent acoustic exposure conditions. In vivo, Evans blue extravasation increased with the reference pressure amplitude and showed higher mean values following TI ultrasound, with a significant between-mode difference at 0.5 MPa. After 0.3-MPa TI sonication, extravasation progressively decreased from 0.5 to 12 h, consistent with a transient BBB permeability increase. Non-Newtonian rheology altered the local shear environment but had limited effects on peak normal stress and porosity within the simulated time window. **Conclusion:** TI ultrasound enhances MB-mediated BBB opening under energy-normalized acoustic exposure conditions while producing a time-limited increase in permeability, supporting its further development for temporally controlled brain delivery.

## Introduction

The blood–brain barrier (BBB), a critical defense mechanism of the central nervous system, constitutes a dynamic interface predominantly composed of brain capillary endothelial cells [1]. This special physiological structure blocks neurotoxic substances, pathogens, and inflammatory factors while precisely regulating brain microenvironment exchange through tight junctions and selective transporters [2]. However, its highly selective permeability prevents more than 98% of small-molecule drugs and nearly all macromolecular therapeutic agents from effectively reaching the brain, thereby hindering therapeutic progress for neurological disorders such as Alzheimer disease and brain tumors [3–5]. Therefore, the ability to modulate BBB permeability is crucial to enable therapeutic agent accumulation for treating such brain disorders.

Focused ultrasound (FUS) has been investigated as a noninvasive approach for modulating BBB permeability. Early studies showed that high-intensity pulsed ultrasound could induce localized BBB disruption, although excessive acoustic exposure was associated with vascular and tissue damage [6]. Subsequent studies further demonstrated localized and reversible BBB opening using magnetic-resonance-guided focused ultrasound [7]. To improve the controllability of BBB opening and reduce the required acoustic exposure, MBs have been introduced to enhance the local mechanical effects of ultrasound.

Numerous studies have demonstrated that FUS combined with microbubbles (MBs) can reversibly open the BBB [8,9]. This is based on the principle that FUS induces MBs in intracranial blood vessels to undergo a series of cavitation effects such as oscillation, expansion, contraction, and collapse. These effects stretch and compress BBB structures in the ultrasound-irradiated region, opening the BBB and enabling drugs or therapeutic molecules to enter the brain [10].

At low acoustic pressure, MBs exhibit stable oscillations that generate radiation forces and localized microstreaming, which can enhance local mass transfer and membrane permeability [11]. When the acoustic pressure is above the inertial cavitation threshold, nonlinear expansion and violent collapse of MBs generate intense mechanical effects as the primary mechanism for ultrasound-MB-mediated BBB opening [12]. However, excessive ultrasound exposure can induce irreversible bioeffects in surrounding tissues, necessitating precise control over treatment parameters.

FUS combined with MBs has been widely investigated as a noninvasive and spatially controllable strategy for transient BBB opening. Studies confirm that BBB opening can be repeatedly and safely achieved across diverse ultrasound parameters [13,14]. Rezai et al. [15] demonstrated the clinical feasibility of noninvasive hippocampal BBB opening in patients with Alzheimer disease. Sierra et al. [16] further demonstrated the feasibility of employing MBs as carriers for targeted delivery of the fluorescent agent 5-dodecanoylaminofluorescein into mouse brains. Lin et al. [17] developed an MB carrier capable of delivering the *GDNF* or *BDNF* gene to the brains of Parkinson disease mice during FUS-induced BBB opening, leading to improved behavioral performance. In addition to advances in therapeutic delivery, practical FUS platforms have been developed to improve targeting precision and experimental accessibility. Hu et al. [18] integrated miniaturized FUS transducers with a stereotactic frame and demonstrated spatially precise BBB opening for targeted drug delivery to the mouse brain. FUS-mediated brain delivery has also been extended beyond conventional systemic administration; Ye et al. [19] demonstrated incisionless and spatially targeted delivery of adeno-associated viral vectors to selected brain regions using FUS-mediated intranasal administration. These developments highlight the importance of combining controllable BBB modulation with accurate spatial targeting for targeted brain delivery. Guo et al. [20] showed that ultrasound-frequency-dependent MB dynamics can modulate BBB permeability and associated inflammatory responses.

Temporal interference (TI) ultrasound is based on the superposition of 2 acoustic components with different frequencies, producing a temporally modulated waveform that can alter MB dynamics. In the present study, the 1.0- and 0.8-MHz components were combined and delivered through the same FUS transducer and acoustic pathway, rather than being spatially superimposed using 2 independently positioned transducers. Therefore, the present implementation can be regarded as a co-propagating dual-frequency form of TI excitation, in which temporal modulation arises from the superposition of the 2 frequency components. Previous studies have shown that dual- or multifrequency excitation can reduce cavitation thresholds and modify Bjerknes forces, interfacial mass transfer, and peak negative pressure, with related effects demonstrated in thrombolysis and tissue ablation [21–25].

Previous studies have established the potential of ultrasound-mediated MB cavitation for BBB modulation; however, computational and in vivo findings have often been reported separately, leaving the relationships among MB dynamics, vessel-wall mechanics, pressure-dependent permeability, and post-sonication recovery incompletely characterized. In this study, we developed a time-dependent, multiphysics-coupled model of the MB–blood vessel system and evaluated its predictions in a mouse model. Normal stress, shear stress, vessel-wall porosity, MB size, and the effects of acoustic pressure were examined under single-frequency and TI excitation. Mesh-independence analysis and a Carreau–Yasuda sensitivity study were performed to assess numerical robustness and the influence of non-Newtonian blood rheology. In vivo Evans blue experiments were then used to compare BBB opening across pressure levels and to characterize the recovery profile from 0.5 to 12 h after 0.3-MPa TI sonication. This integrated approach was designed to determine whether TI ultrasound could enhance BBB opening under energy-normalized acoustic exposure conditions while preserving a transient permeability window. The overall study framework is illustrated in Fig. 1.

**Fig. 1.**
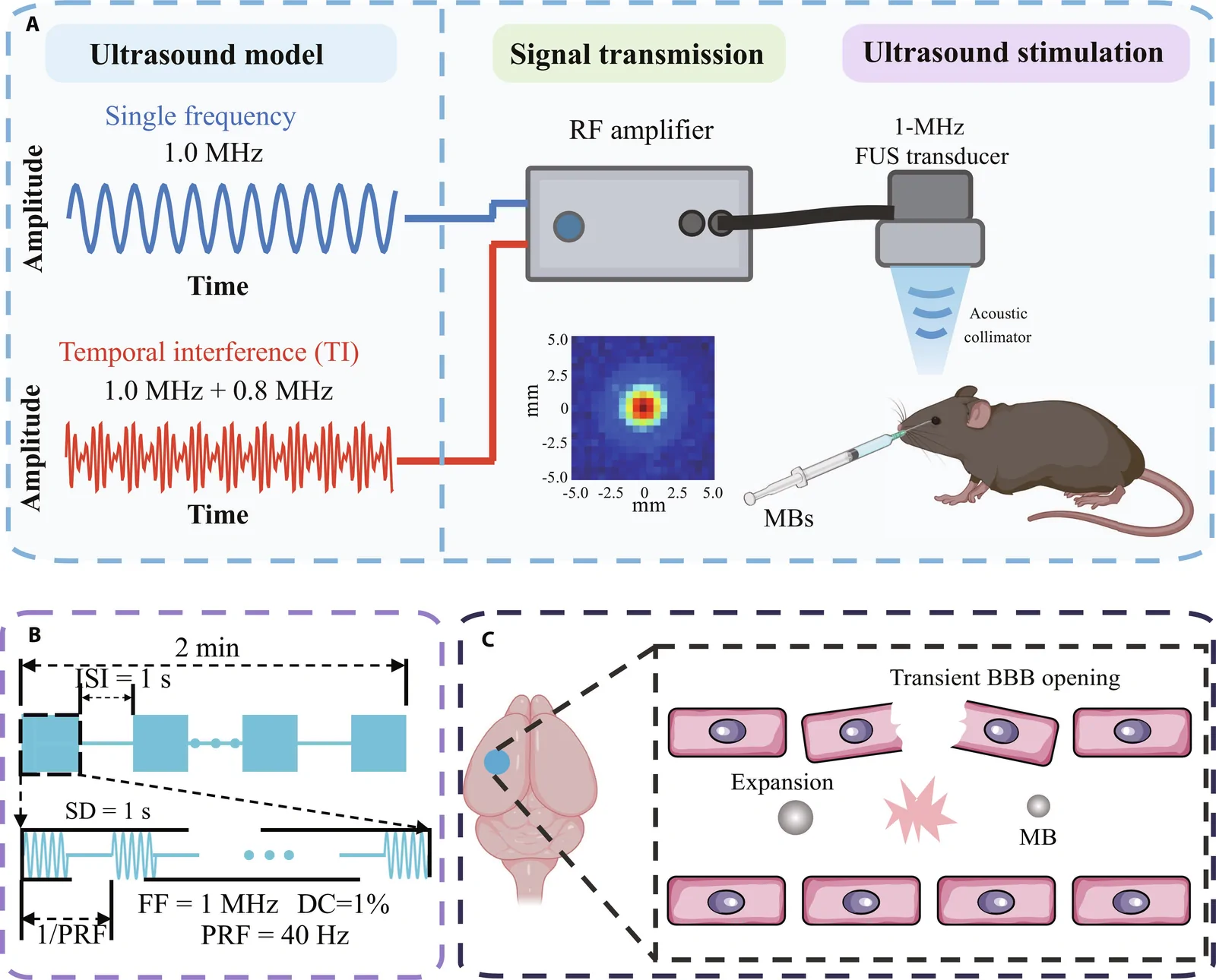
Experimental configuration and schematic of ultrasound-mediated blood–brain barrier (BBB) opening. (A) Schematic illustration of BBB opening induced by ultrasound combined with microbubbles (MBs). RF, radiofrequency; FUS, focused ultrasound. (B) Ultrasound stimulation parameters. PRF, pulse repetition frequency; DC, duty cycle; ISI, interstimulus interval; SD, stimulation duration; FF, fundamental frequency. (C) Schematic illustration of microbubble oscillation and transient BBB opening in cerebral microvessels.

## Results

### Model validation

To validate the time-varying model of the MB–vessel system, the MB radius was computed using Eq. (1) with parameters matching the reference study [26]. For an initial MB radius, the analytical solution and numerical solutions exhibited similar trends. Minor discrepancies are attributed to finite numerical precision and to the omission of shell viscous effects. These comparisons are presented in Fig. 2A.

$$\rho \left(R\ddot{R}+\frac{3}{2}{\overset{\cdot }{R}}^{2}\right)=P_{g0}\;{\left(\;\frac{R_{i0}^{3}}{R_{i}^{3}}\;\right)}^{\gamma }-2\frac{\sigma_{g}}{R}-4k_{s}\frac{\overset{\cdot }{R}}{R^{2}}-4\mu_{f}\frac{\overset{\cdot }{R}}{R}-P_{u}-P_{0}$$ (1)

**Fig. 2.**
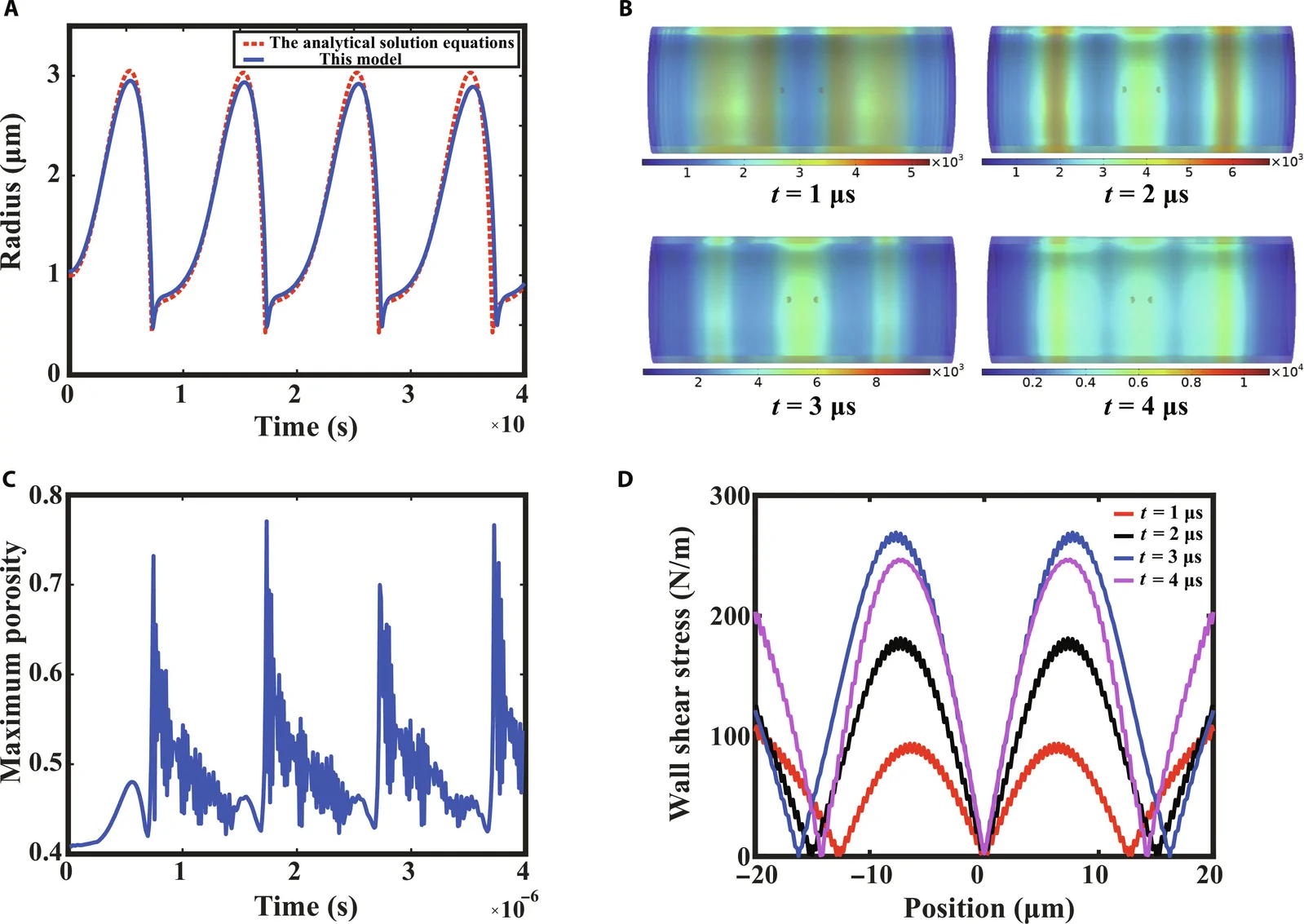
Model validation and baseline vessel-wall mechanical responses under single-frequency ultrasound. (A) Comparison of the simulated MB-radius response with the analytical reference; (B) vessel-wall normal stress at 1, 2, 3, and 4 μs; (C) vessel-wall porosity at different time points; and (D) vessel-wall shear-stress distribution.

Mesh independence was assessed to minimize discretization-related bias and identify a converged mesh for subsequent simulations. Six free-triangular meshes with different maximum element sizes were constructed, and the maximum vessel-wall porosity at *t* = 0.5 μs was selected as the convergence metric. As summarized in Table 1, the calculated maximum porosity varied only slightly among the 6 cases and approached a stable range with mesh refinement. Further refinement markedly increased the number of elements and computational cost, whereas excessively coarse meshes reduced the spatial resolution of stress and porosity maps. Case 3, with a maximum element size of 0.4 μm and 38,231 triangular elements, was therefore selected as the standard mesh. For reference, the acoustic wavelength at 1 MHz was approximately 1.5 mm, substantially larger than the selected local mesh scale.

**Table 1.** Mesh-independence verification for cases 1 to 6

Case	1	2	3	4	5	6
Maximum element size (μm)	0.2	0.3	0.4	1.6	2.96	4.24
Number of triangular elements	92,387	54,901	38,231	11,463	9,497	7,963
Maximum porosity	0.4713	0.4719	0.4713	0.4697	0.4690	0.4688

### Vessel-wall normal stress

Ultrasound-mediated MB oscillation generates high-frequency normal stress within the vascular lumen. This stress propagates radially through the vessel wall, imparting transient tensile forces on endothelial cells and mechanically widening inter-endothelial junctions, thereby providing a mechanical basis for increased BBB permeability. As shown in Fig. 2B, under single-frequency excitation, MB cavitation generated mechanical stress on the vessel wall, reaching magnitudes up to 10^4^ Pa within 4 μs. At 1, 2, 3, and 4 μs, the corresponding normal stresses were 5.32 × 10^3^ Pa, 6.85 × 10^3^ Pa, 9.82 × 10^3^ Pa, and 1.10 × 10^4^ Pa, respectively.

### Vessel-wall porosity

Vessel-wall porosity was used as a model-based indicator related to BBB permeability. Under physiological conditions, tight junctions between brain capillary endothelial cells strongly restrict paracellular transport. Ultrasound-driven MB cavitation can transiently deform the vascular wall and alter the local barrier structure. As shown in Fig. 2C, modeled porosity increased from a baseline of 0.406 to a peak of 0.542 at 1 μs, followed by values of 0.522, 0.460, and 0.536 at 2, 3, and 4 μs, respectively. These results demonstrate a rapid, time-dependent increase in modeled vessel-wall porosity during MB oscillation.

### Vessel-wall shear stress

Shear stress is a key factor regulating BBB permeability. The cavitation of MBs driven by ultrasound generates instantaneous, localized high shear stress on the vascular endothelium. Such localized shear loading may transiently perturb endothelial barrier integrity and thereby contribute to increased BBB permeability. A 40-μm vessel segment was modeled to quantify spatial shear-stress distribution along the vessel wall–blood interface under single-frequency ultrasound. As shown in Fig. 2D, shear stress peaked near the oscillating MBs (∼10 kPa).

### Non-Newtonian blood rheology

The local non-Newtonian rheological response was evaluated at an acoustic pressure of 0.3 MPa. Compared with the relatively mild response at 0.1 MPa, this higher pressure produced more pronounced flow disturbances and shear effects. As shown in Fig. 3A, the apparent blood viscosity decreased rapidly at the onset of ultrasound exposure and then remained close to a stable level, with small fluctuations and transient peaks. This behavior indicates that the elevated acoustic pressure increased the local shear rate and induced a clear shear-thinning response. The shear stress–shear rate trajectory in Fig. 3B exhibited a nonlinear loop-like pattern, reflecting the strongly unsteady local flow during ultrasound exposure. These results suggest that non-Newtonian rheology may modify local viscous damping, shear-stress distribution, and the mechanical stimulus applied to endothelial cells during BBB opening.

**Fig. 3.**
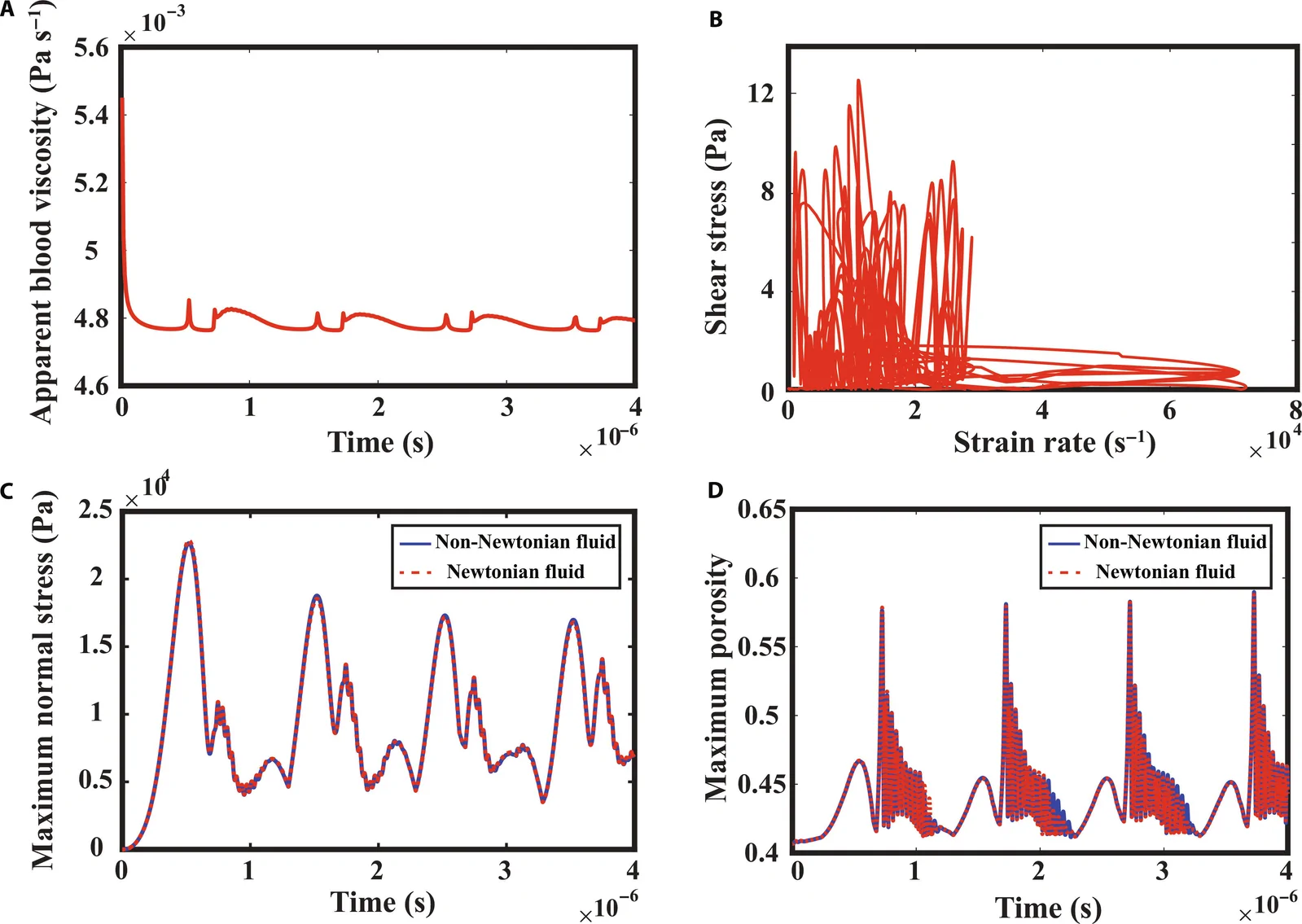
Non-Newtonian blood rheology and its influence on vessel-wall response during ultrasound-mediated BBB opening. (A) Temporal variation in apparent blood viscosity. (B) Shear stress–shear rate relationship. (C) Temporal evolution of maximum vessel-wall normal stress predicted by the Newtonian and non-Newtonian blood models. (D) Temporal evolution of maximum vessel-wall porosity predicted by the Newtonian and non-Newtonian blood models.

### Influence of blood rheology on vessel-wall response

The lower panels of Fig. 3 compare the temporal evolution of maximum normal stress and maximum porosity obtained using the Newtonian and non-Newtonian blood models. Within the simulated time window, the Newtonian and non-Newtonian models exhibited nearly identical trends in maximum normal stress, with comparable peak timings and overall oscillatory patterns and only minor differences in local amplitude (Fig. 3C). Maximum porosity also exhibited comparable periodic increases and decreases in both models, and the difference remained limited (Fig. 3D). Thus, although the non-Newtonian formulation alters the local apparent viscosity, shear rate, and shear-stress distribution, its influence on the global maxima of normal stress and porosity remained limited under the present acoustic conditions and within the simulated time window.

### Maximum vessel-wall porosity and normal stress

At 0.4 MPa and 0.5 MPa, MBs approached collapse in less than 1 μs. To distinguish the temporal responses associated with stable and inertial cavitation, the vessel-wall response was analyzed over the first 0.5 μs under single-frequency excitation. Figure 4 shows the temporal evolution of maximum vessel-wall porosity and normal stress at 0.3, 0.4, and 0.5 MPa. Both quantities increased monotonically over this interval. The peak wall normal stresses were 2.674 × 10^4^ Pa, 6.431 × 10^4^ Pa, and 1.196 × 10^5^ Pa, while the corresponding maximum porosities were 0.471, 0.565, and 0.723, respectively.

**Fig. 4.**
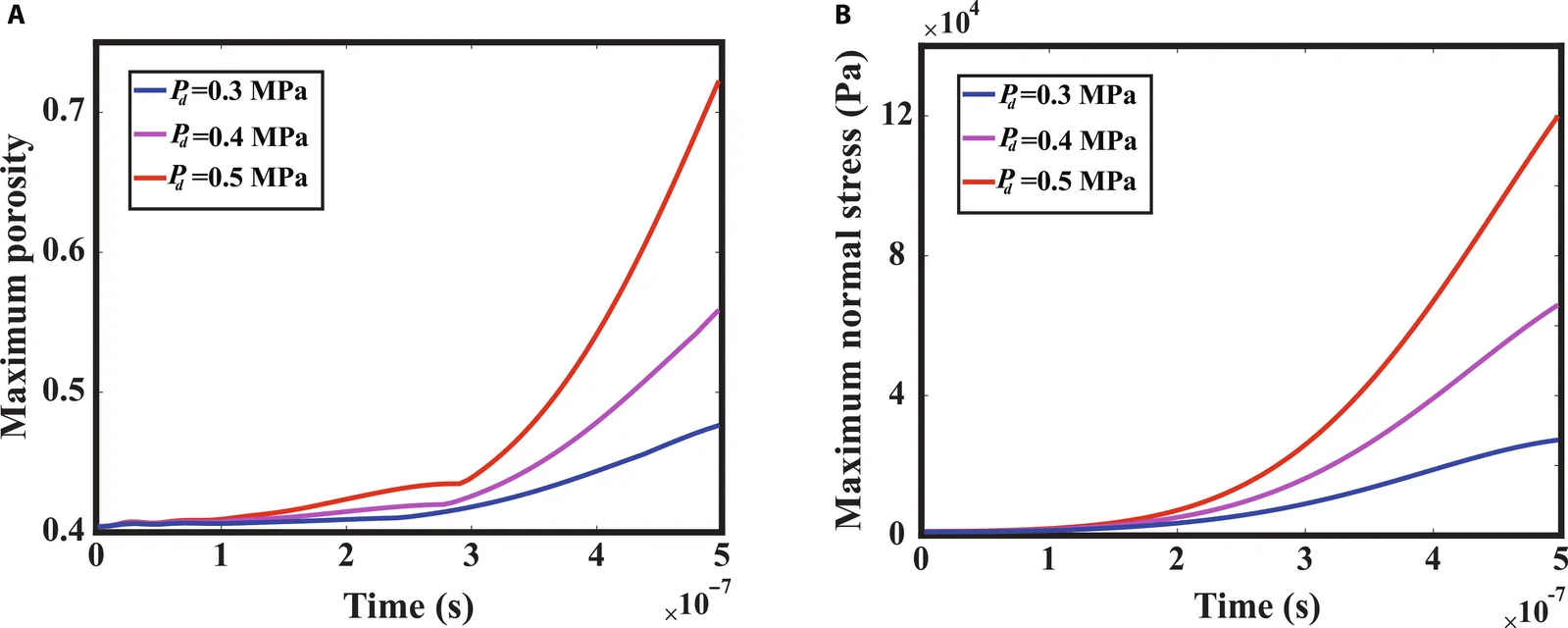
Changes in maximum vessel-wall (A) porosity and (B) normal stress over time at different acoustic pressures.

### TI ultrasound stimulation

The effect of TI ultrasound (1.0 + 0.8 MHz) on vessel-wall mechanical response was investigated and compared with single-frequency excitation, with all other parameters held constant. TI stimulation enhanced MB instability, advancing the onset of collapse; at 0.927 μs, MBs approached inertial collapse. Concurrently, the stronger mechanical confinement imposed by the vessel wall under TI excitation progressively deformed the MBs into ellipsoidal shapes, ultimately exhibiting pronounced deformation immediately before collapse (Fig. 5A). Overall, TI ultrasound promoted earlier inertial collapse than single-frequency exposure, consistent with previous numerical findings [24].

**Fig. 5.**
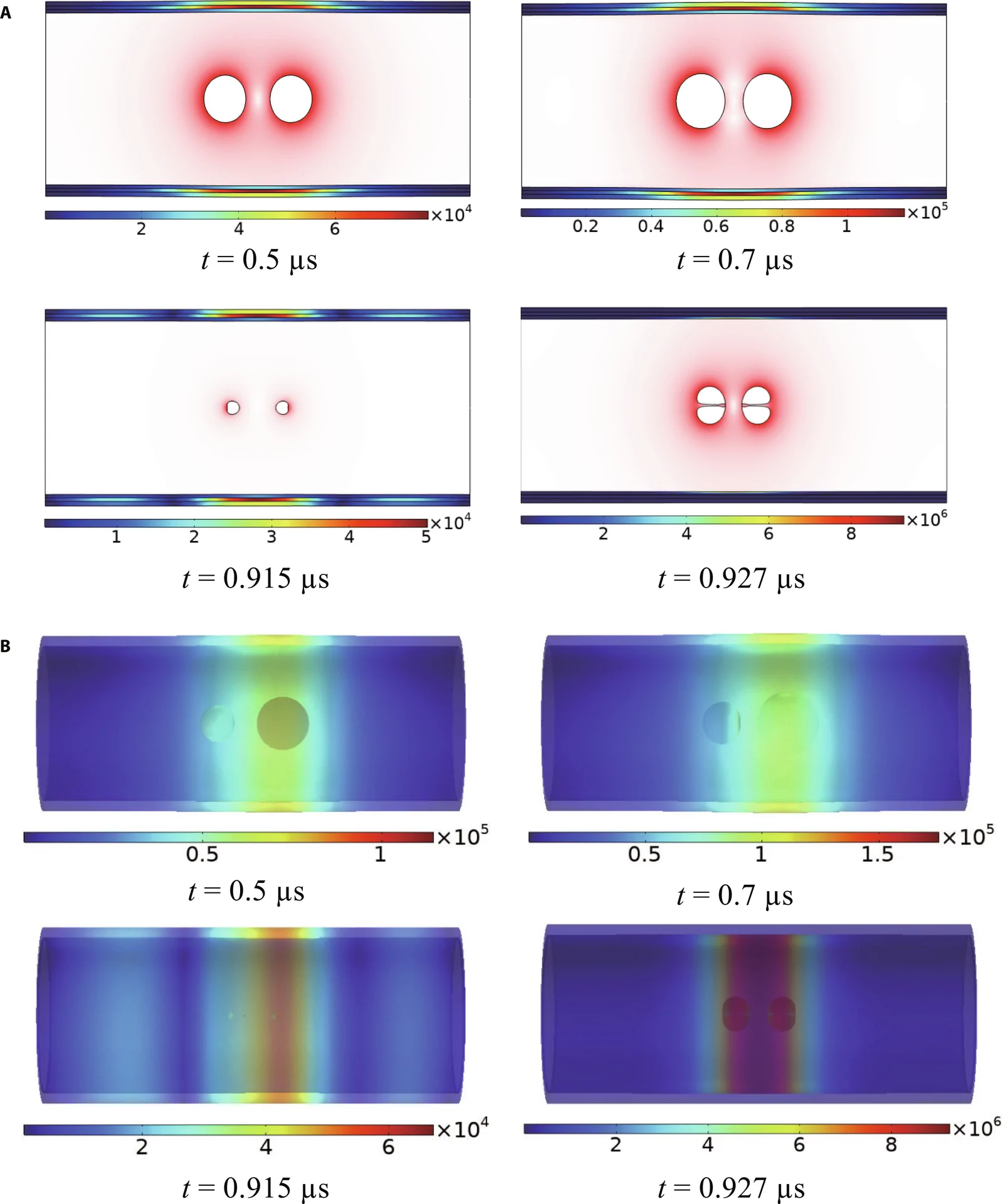
Effects of temporal interference (TI) excitation and initial MB radius on cavitation dynamics and vessel-wall stress. (A) Temporal evolution of MB deformation and collapse under 1.0 + 0.8 MHz TI ultrasound. (B) Size-dependent cavitation behavior under single-frequency excitation, showing different collapse dynamics for MBs with different initial radii.

### Effect of MB radius

To examine the influence of MB size, simulations were performed with initial radii of 1.0 and 1.4 μm, corresponding to diameters of 2.0 and 2.8 μm, respectively. These values are within the reported size distribution of SonoVue MBs and are close to their mean diameter of approximately 2.5 μm. As shown in Fig. 5B, under single-frequency excitation, 1.0-μm MBs underwent prompt transient cavitation, whereas 1.4-μm MBs reached the collapse threshold later. This size-dependent response is consistent with experimental and theoretical evidence that cavitation thresholds depend strongly on the initial MB size [27,28]. These findings underscore the importance of considering MB size distributions in both simulations and in vivo applications, as heterogeneous MB populations can alter cavitation dynamics and, consequently, BBB permeability.

### Enhanced BBB permeability and recovery following TI ultrasound

To test the computational prediction that TI ultrasound modulates the BBB more efficiently than single-frequency ultrasound under energy-equivalent exposure conditions, we performed in vivo experiments in mice. As shown in Fig. 6A, single-frequency or TI ultrasound was delivered to the left cerebral hemisphere at reference pressure amplitudes $P_{d}$ of 0.3, 0.4, and 0.5 MPa 1 min after retro-orbital administration of MBs.

**Fig. 6.**
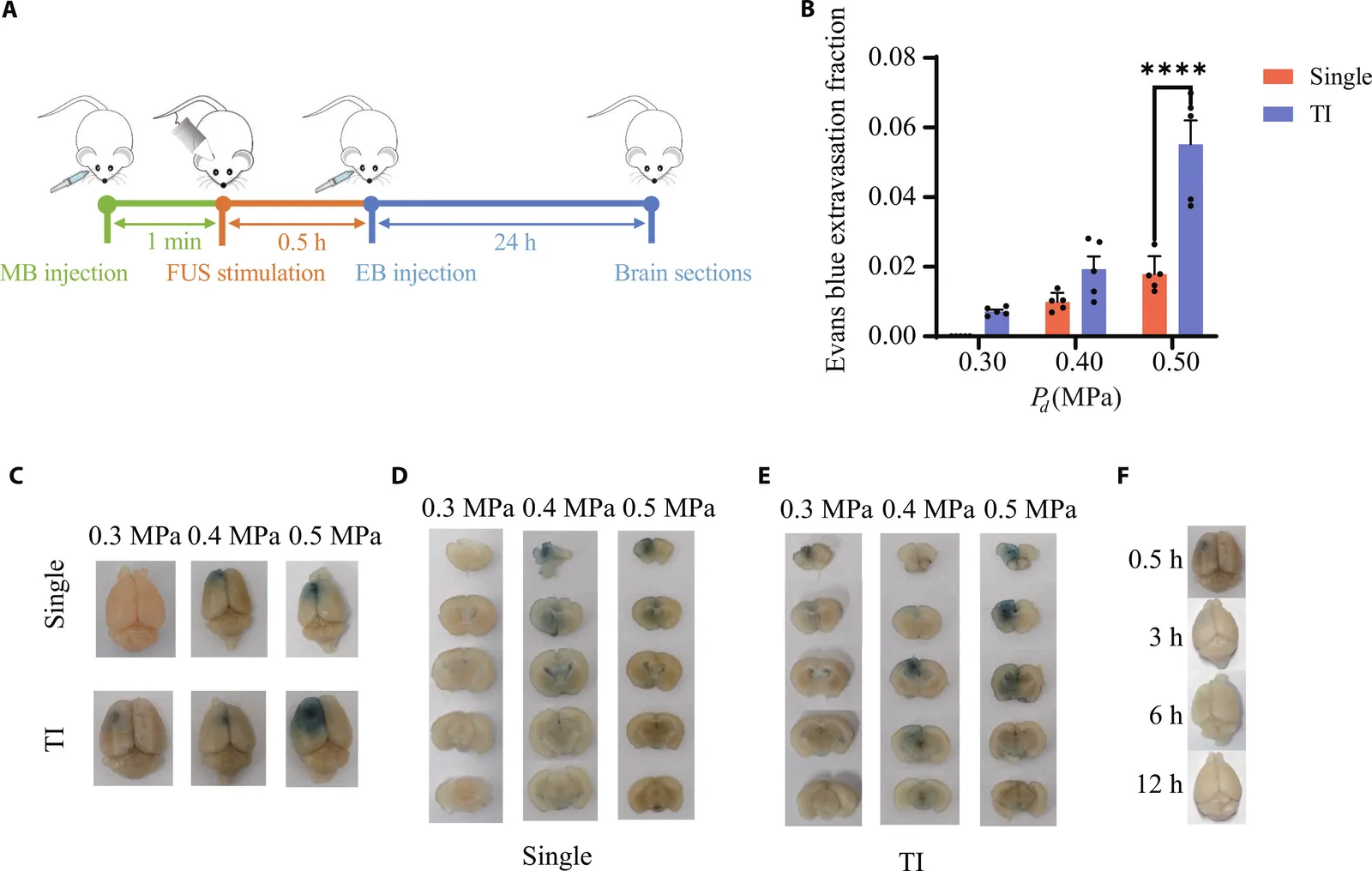
In vivo validation of BBB opening and recovery using TI and single-frequency ultrasound combined with MBs. (A) Experimental protocol. EB, Evans blue. (B) Quantitative analysis of Evans blue extravasation. Individual data points represent individual animals, and bars indicate mean ± SEM (*n* = 5 per group). Statistical significance was assessed by 2-way ANOVA followed by Šídák’s multiple-comparisons test. ****P < 0.0001. (C to E) Representative whole-brain and coronal-section images at $P_{d}=0.3\;\text{to}\;0.5\;\mathrm{MPa}$, showing greater BBB permeability in the TI group (1.0 + 0.8 MHz) than in the single-frequency group (1.0 MHz). (F) Representative whole-brain images obtained 0.5, 3, 6, and 12 h after TI sonication at $P_{d}=0.3\;\mathrm{MPa}$ (*n* = 3 per time point), showing a time-dependent decline in Evans blue extravasation, consistent with a progressive reduction in BBB permeability.

As shown in Fig. 6B to E, TI ultrasound produced broader and more intense Evans blue extravasation in the target region than single-frequency ultrasound at the same $P_{d}$. Because the 2 stimulation modes were normalized to the same root mean square (RMS) pressure and time-averaged squared pressure at each $P_{d}$, this difference cannot be attributed simply to a greater nominal time-averaged acoustic energy input. BBB permeability increased with $P_{d}$ under both stimulation modes, but the increase was more pronounced with TI ultrasound. Two-way analysis of variance (ANOVA) indicated a significant stimulation mode × $P_{d}$ interaction. Šídák’s multiple-comparisons test showed that Evans blue extravasation was significantly greater with TI ultrasound than with single-frequency ultrasound at $P_{d}=0.5\;\mathrm{MPa}\;$(*P* < 0.0001), whereas the differences at 0.3 and 0.4 MPa did not reach statistical significance. These in vivo findings are consistent with the model prediction that the temporally modulated TI waveform enhances MB instability and accelerates cavitation collapse.

To examine recovery after low-pressure TI sonication, Evans blue extravasation was assessed at 0.5, 3, 6, and 12 h after exposure at $P_{d}=0.3\;\mathrm{MPa}$. As shown in Fig. 6F, representative Evans blue images showed the greatest extravasation at 0.5 h, followed by a progressive decrease at 3 and 6 h and only limited residual extravasation at 12 h. This pattern indicates a time-dependent reduction in BBB permeability under the present stimulation conditions. Because the assessment was based on representative Evans blue images at discrete time points, these data characterize the approximate time course of permeability reduction but do not establish the precise time of complete BBB closure.

Taken together, the pressure-response and recovery findings suggest that TI ultrasound can create a comparatively strong but time-limited permeability window at low reference pressure amplitude $P_{d}$. This combination may be advantageous for brain delivery because it could improve target access while limiting the duration of barrier disruption. Nevertheless, a lower reference pressure amplitude should not be interpreted as evidence of safety on its own; quantitative longitudinal permeability measurements, cavitation monitoring, and histological assessment will be needed to define the therapeutic window more precisely.

## Discussion

This study combined a time-dependent MB–vessel model with in vivo experiments to examine how TI ultrasound influences cavitation behavior, vessel-wall mechanics, BBB opening efficiency, and post-sonication recovery. The principal finding is that TI excitation produced greater BBB modulation than 1.0-MHz single-frequency ultrasound under energy-equivalent acoustic exposure conditions. At a given reference pressure amplitude $P_{d}$, the single-frequency and TI waveforms had the same RMS pressure and time-averaged squared pressure, whereas their instantaneous temporal waveforms differed. The enhanced response observed with TI ultrasound therefore cannot be explained simply by an increase in nominal time-averaged acoustic energy. In addition, TI exposure at $P_{d}=0.3\;\mathrm{MPa}$ generated a permeability increase that diminished progressively over time. Together, these findings indicate that temporal modulation of the acoustic waveform can enhance MB-mediated BBB modulation under controlled, energy-normalized exposure conditions.

The simulations provide a mechanical explanation for this effect. Ultrasound-driven MB oscillation generated both normal and shear stresses on the vessel wall and increased the modeled wall porosity. As acoustic pressure increased, the peak normal stress and porosity rose markedly, indicating a direct relationship between cavitation intensity and the mechanical loading associated with BBB modulation. This pressure dependence is consistent with the established role of MB cavitation in ultrasound-mediated BBB opening [12,29]. The present model further links these cavitation dynamics to transient changes in vessel-wall porosity, thereby connecting bubble-scale behavior with a barrier-level mechanical response. Beyond their role in modulating BBB permeability, MB-induced mechanical effects may also influence fluid transport in the perivascular environment. Ye et al. [30] demonstrated that ultrasound-driven MB expansion and contraction can exert cyclic pushing and pulling forces on the vessel wall, generating a mechanical pumping effect that enhances tracer transport along perivascular spaces and promotes transport into the surrounding interstitial space. Although perivascular or glymphatic transport was not directly evaluated in the present study, these findings suggest that the vessel-wall stresses and deformation associated with MB oscillation may have broader implications for vascular perivascular transport in addition to transient BBB permeability changes.

TI excitation altered this response by promoting MB instability and advancing the onset of collapse compared with single-frequency excitation. This observation is consistent with previous numerical studies showing that multifrequency excitation can reduce the inertial cavitation threshold. In the present simulations, the combined 1.0- and 0.8-MHz excitation enhanced MB deformation and collapse, which was accompanied by increased vessel-wall mechanical loading. The greater BBB modulation observed with TI stimulation is therefore consistent with altered cavitation dynamics rather than a simple increase in nominal acoustic pressure [21,24].

The in vivo Evans blue findings support this interpretation. BBB permeability increased with acoustic pressure under both stimulation modes, with higher mean Evans blue extravasation in the TI groups; the between-mode difference reached statistical significance at 0.5 MPa. Importantly, after 0.3-MPa TI sonication, Evans blue extravasation was strongest at 0.5 h and progressively decreased at 3, 6, and 12 h. This pattern indicates that the permeability enhancement was transient under the present conditions and supports the presence of a permeability window that may be relevant to the timing of therapeutic administration [31]. However, because the recovery experiment was based on representative Evans blue images, the present data define the direction and approximate time course of the reduction in BBB permeability rather than the exact time of complete barrier closure. Previous studies have similarly shown that the duration and magnitude of BBB permeability depend on cavitation dose, MB exposure, tracer size, and post-sonication assessment time.

The non-Newtonian sensitivity analysis also clarifies the role of blood rheology in the coupled response. The Carreau–Yasuda formulation revealed pronounced local shear-thinning and a nonlinear shear stress–shear rate relationship during ultrasound exposure. Nevertheless, the maximum normal stress and porosity remained close to those obtained with the Newtonian model within the simulated time window. Thus, under the acoustic conditions examined here, non-Newtonian behavior appears to modify the local viscous and shear environment more strongly than the global peak vessel-wall response. These results suggest that a Newtonian approximation may be adequate for the present comparative simulations while also identifying local rheology as a relevant factor for more detailed endothelial-scale analyses.

Several limitations should be considered. First, BBB recovery was evaluated qualitatively from Evans blue images at selected time points; quantitative longitudinal permeability measurements are needed to determine the closure kinetics more precisely. Second, cavitation activity was inferred from the coupled model rather than measured directly in vivo. Future studies should incorporate passive acoustic monitoring to characterize MB cavitation activity and enable feedback-controlled exposure [32,33]. Beyond cavitation detection and intensity monitoring, spatial localization of cavitation emissions may also be important for transcranial applications, as it could help confirm target engagement and identify potential off-target cavitation events. Notably, low-channel-count approaches have demonstrated the feasibility of 3-dimensional transcranial MB cavitation localization using only 4 acoustic sensors, providing a relatively simple strategy for spatially resolved cavitation monitoring [34]. Third, transcranial targeting accuracy is another methodological consideration. Although stereotactic coordinates and hydrophone measurements were used to define the sonication region, the spatial offset between the intended target and the actual intracranial focus was not directly quantified. Skull-induced aberration, variations in acoustic coupling, and inter-animal anatomical variability may affect focal position and geometry. Previous studies have shown that targeting accuracy can be evaluated by combining hydrophone measurements, in vivo localization, and subject-specific acoustic simulations [35]. Fourth, histological and neuroinflammatory assessments are required to define the safety margin associated with the enhanced mechanical effects of TI stimulation because FUS-mediated BBB opening can induce pressure- and exposure-dependent cellular and immune responses [29,36]. Finally, the present model simplifies the vascular geometry and material behavior and does not capture the full heterogeneity of microvessels, MB size distributions, or tissue-specific acoustic propagation.

Overall, the combined computational and experimental results indicate that TI ultrasound can enhance MB-mediated BBB opening at comparatively low acoustic pressure and generate a time-limited permeability window. The work provides a mechanistic framework for selecting acoustic parameters and coordinating therapeutic administration with the period of increased BBB permeability. The growing clinical use of ultrasound-mediated BBB opening for therapeutic delivery further highlights the importance of precise control over permeability magnitude and duration [37,38]. Further quantitative permeability, cavitation-monitoring, and histological studies will be important for defining the therapeutic window and evaluating the translational potential of TI ultrasound.

## Materials and Methods

### Fluid–solid coupling

As illustrated in Fig. 1C, a time-varying model of the MB–vessel system was established to elucidate the mechanism of FUS-induced, MB-mediated BBB opening. The vessel was defined with the following geometric parameters: length *L* = 80 μm, radius *Rv* = 15 μm, and wall thickness *h* = 2 μm (comprising intima: 0.6 μm, media: 0.6 μm, and adventitia: 0.8 μm), with the MB centered in the vessel. Fluid–structure interaction theory was employed to study the mutual influence between blood flow and vessel walls. Blood was initially treated as an incompressible Newtonian fluid, and its time-dependent evolution was governed by the mass conservation equation and the Navier–Stokes equations, expressed as follows [39]:

$$\frac{\partial \rho }{\partial t}+\nabla \cdot \left(\rho \vec{u}\right)=0$$ (2)

$$\rho \left(\frac{\partial \vec{u}}{\partial t}+\left(\vec{u}\cdot \nabla \right)\vec{u}\right)-\nabla \cdot \left(-p\vec{I}+\mu_{f}\left(\nabla \vec{u}+\nabla {\vec{u}}^{T}\right)\right)=0$$ (3)

Equation (2) represents the principle of mass conservation, and Eq. (3) describes the dynamic relationship between fluid acceleration, velocity, and pressure. Here, *p*, *ρ*, and *μ_f_* denote pressure, density, and dynamic viscosity of the fluid, respectively; *I* is the identity tensor and *u* is the velocity vector. To determine the stress magnitude and spatial distribution within the MB–vessel system under hemodynamic loading, linear elasticity theory was adopted within a small-deformation framework, assuming a linear stress–strain constitutive relationship. The vessel tissue was modeled as an isotropic, linear-elastic material, with its mechanical behavior governed by the following equation [40]:

$$\rho_{s}\frac{\partial^{2}\vec{u_{s}}}{\partial t^{2}}-\nabla^{T}\vec{\sigma }=\vec{b_{s}}$$ (4)

$$\vec{u_{s}}=\left[\begin{array}{c} u_{1}^{s}\\ u_{2}^{s}\\ u_{3}^{s} \end{array}\right]$$ (5)

$$\vec{b_{s}}=\left[\begin{array}{c} b_{1}^{s}\\ b_{2}^{s}\\ b_{3}^{s} \end{array}\right]$$ (6)

$$\nabla =\left[\begin{array}{c} \begin{array}{ccc} \frac{\partial }{\partial x_{1}} & 0 & 0\\ 0 & \frac{\partial }{\partial x_{2}} & 0\\ 0 & 0 & \frac{\partial }{\partial x_{3}} \end{array}\\ \begin{array}{ccc} \frac{\partial }{\partial x_{2}} & \frac{\partial }{\partial x_{1}} & 0\\ \frac{\partial }{\partial x_{3}} & 0 & \frac{\partial }{\partial x_{1}}\\ 0 & \frac{\partial }{\partial x_{3}} & \frac{\partial }{\partial x_{2}} \end{array} \end{array}\right]$$ (7)

Here, $\rho_{s}$ denotes the solid density, $b_{s}$ denotes the body-force vector, ∇ denotes the nabla operator, and $\vec{u_{s}}$ and $\vec{\sigma }$ correspond to the solid displacement vector and stress tensor, respectively. The stress tensor $\vec{\sigma }$ is derived from the constitutive equation of the isotropic, linear-elastic material.

The Green–Lagrange strain tensor $\vec{\varepsilon }$ and the stress tensor $\vec{\sigma }\;$are expressed in matrix form:

$$\vec{\varepsilon }={\left[\varepsilon_{11}^{s}\;\varepsilon_{22}^{s}\;\varepsilon_{33}^{s}\;2\varepsilon_{12}^{s}\;2\varepsilon_{13}^{s}\;2\varepsilon_{23}^{s}\right]}^{T}$$ (8)

$$\vec{\sigma }={\left[\sigma_{11}^{s}\;\sigma_{22}^{s}\;\sigma_{33}^{s}\;\sigma_{12}^{s}\;\sigma_{13}^{s}\;\sigma_{23}^{s}\right]}^{T}$$ (9)

The displacement–strain relationship of the solid tissue is governed by the following kinematic equation:

$$\vec{\varepsilon }=\nabla \vec{u_{s}}$$ (10)

For isotropic materials, the constitutive relationship between strain and stress is

$$\vec{\sigma }=\vec{D}\vec{\varepsilon }$$ (11)

where the matrix $\vec{D}$ is defined as

$$\vec{D}=\left[\begin{array}{cc} \begin{array}{c} \begin{array}{ccccc} & \lambda +2\mu & \lambda & \lambda & \\ & \lambda & \lambda +2\mu & \lambda & \\ & \lambda & \lambda & \lambda +2\mu & \\ 0 & & 0 & & 0\\ 0 & & 0 & & 0\\ 0 & & 0 & & 0 \end{array} \end{array} & \begin{array}{c} \begin{array}{ccc} 0 & 0 & 0\\ 0 & 0 & 0\\ 0 & 0 & 0 \end{array}\\ \begin{array}{ccc} \mu & 0 & 0\\ 0 & \mu & 0\\ 0 & 0 & \mu \end{array} \end{array} \end{array}\right]$$ (12)

λ and μ (the Lamé coefficients) are calculated from Young’s modulus E and Poisson’s ratio ν using

$$\lambda =\frac{\nu E}{\left(1+\nu \right)\left(1-2\nu \right)}$$ (13)

$$\mu =\frac{E}{2\left(1+\nu \right)}$$ (14)

The fluid–solid boundary satisfies the following continuity conditions:

$$\vec{d_{\mathrm{ff}}}=\vec{d_{\mathrm{ss}}}$$ (15)

$$\vec{\sigma_{\mathrm{ff}}}\cdot \vec{m_{\mathrm{ff}}}=\vec{\sigma_{\mathrm{ss}}}\cdot \vec{m_{\mathrm{ss}}}$$ (16)

$$\vec{u_{\mathrm{ff}}}=\vec{u_{\mathrm{ss}}}$$ (17)

Equations (15) to (17) enforce continuity of displacement, velocity, and stress at the fluid–solid interface, where d, u, and σ denote displacement, velocity, and stress tensor, respectively, and m is the outward unit normal vector. Subscripts “ff” and “ss” refer to the fluid and solid domains, respectively.

### Bubble dynamics

The pressure within cavitation MBs is governed by the van der Waals equation of state [41]:

$$P_{g}=P_{g0}\;{\left(\;\frac{R_{i0}^{3}}{R_{i}^{3}}\;\right)}^{\gamma }-4k_{s}\frac{{\overset{\cdot }{R}}_{i}}{R_{i}^{2}}$$ (18)

$$P_{g0}=P_{0}+\frac{2\sigma_{g}}{R_{0}}-P_{v}$$ (19)

Here, $P_{g}$ denotes the gas pressure inside the cavitation MB at time *t*; $R_{i0}$, the initial radius of the *i*-th MB; γ, the gas polytropic index; and $k_{s}$, the dilatational viscosity (viscous effects of the MB shell are neglected). Under FUS excitation, the cavitation MB boundary is governed by the following equations:

$$u\left(\;R\;\right)=\overset{\cdot }{R}$$ (20)

$$\left(\left(\;P_{g}-p\;\right)I+\mu_{f}\;\left(\nabla \cdot u+\nabla u^{T}\;\right)\right)\cdot q=2\sigma_{g}\mathrm{kq}$$ (21)

Here, q and k denote the unit normal vector and local curvature of the MB surface, respectively. The ultrasound is defined as

Single-frequency: $P_{u}=P_{d}\sin\left(2\pi \mathrm{ft}\right)$; TI: $P_{u}=\frac{P_{d}}{\surd 2}\sin\left(2\pi f_{1}t\right)+\frac{P_{d}}{\surd 2}\sin\left(2\pi f_{2}t\right)$

Here, $P_{d}$ denotes the reference acoustic-pressure amplitude used to normalize the acoustic exposure between the 2 stimulation modes. For single-frequency excitation, the time-averaged squared pressure is ${P_{u}}^{2}=\frac{{P_{d}}^{2}}{2}$. For TI excitation, each frequency component has an amplitude of $\frac{P_{d}}{\surd 2}$, and the cross term between the 2 frequency components averages to zero over the exposure interval. Therefore, ${P_{u}}^{2}=\frac{{P_{d}}^{2}}{4}+\frac{{P_{d}}^{2}}{4}=\frac{{P_{d}}^{2}}{2}$, yielding the same RMS pressure, $P_{\mathrm{RMS}}=\frac{P_{d}}{\surd 2}$, as the single-frequency condition. Thus, comparisons between single-frequency and TI excitation at the same $P_{d}$ were performed under energy-equivalent acoustic exposure conditions rather than peak-pressure-matched conditions. The instantaneous peak of the composite TI waveform was not used as the normalization criterion.

During MB oscillation, the secondary acoustic interaction between the 2 MBs was calculated as follows [42]:

$$P_{\mathrm{rad}1}=\frac{\rho }{D}\frac{d}{\mathrm{dt}}\left(\;\overset{\cdot }{R_{2}}R_{2}^{2}\;\right)$$ (22)

$$P_{\mathrm{rad}2}=\frac{\rho }{D}\frac{d}{\mathrm{dt}}\left(\;\overset{\cdot }{R_{1}}R_{1}^{2}\;\right)$$ (23)

To characterize the effect of MB movement on vessel-wall permeability, the porosity of the vessel wall is described by the following relation [43]:

$$\varepsilon_{p}=\text{exp}\left(b_{p}\left(P-P_{0}\right)\right)$$ (24)

where $P_{0}$ and P denote the initial pressure and the wall pressure at time *t*, respectively. The value of $b_{p}$ is 9 MPa^−1^ and *P*_0_ = 0.1 MPa. The parameter values are listed in Table 2.

**Table 2.** Values of parameters

Name	Parameters	Value (unit)
Gas polytropic	γ	1.07
Saturated vapor pressure	$P_{v}$	2,330 Pa
The initial radius of the MB	$R_{0}$	1 μm
Gas–liquid surface tension	$\sigma_{g}$	0.056 N m^−1^
The density of blood	ρ	1,059 kg m^−3^
Hemodynamic viscosity	$\mu_{f}$	0.0035 Pa·s
The ambient pressure	$P_{0}$	1.013 × 10^5^ Pa
Fundamental frequency	f	1.0 × 10^6^ Hz

The solid-tissue material parameters used in this study [44,45] are listed in Table 3.

**Table 3.** Solid-tissue-related material parameters

Parameters	Density (kg m^−3^)	Poisson’s ratio	Young’s modulus (Pa)
Intima	1,000	0.49	2 × 10^6^
Media	1,000	0.49	6 × 10^6^
Adventitia	1,000	0.49	4 × 10^6^

### Non-Newtonian blood rheology model

In the baseline simulations, blood was represented as a constant-viscosity fluid. At the microvascular scale, however, ultrasound-induced velocity gradients may produce a marked shear-rate dependence. To evaluate the influence of blood rheology on the local flow field and vessel-wall mechanics, a supplementary non-Newtonian analysis was performed using the Carreau–Yasuda model. Accordingly, the constant dynamic viscosity $\mu_{f}$ in Eq. (3) was replaced by the shear-rate-dependent apparent viscosity $\eta \left(\overset{\cdot }{\gamma }\right)$, expressed as

$$\eta \left(\overset{\cdot }{\gamma }\right)=\eta_{\infty }+\left(\eta_{0}-\eta_{\infty }\right){\left[1+{\left(\lambda \overset{\cdot }{\gamma }\right)}^{a_{\mathrm{cy}}}\right]}^{\frac{n_{\mathrm{cy}}-1}{a_{\mathrm{cy}}}}$$ (25)

where $\eta_{0}$ and $\eta_{\infty }$ denote the zero- and infinite-shear-rate viscosities, respectively; λ is the relaxation time constant, *n* is the power-law index, and *a* is the transition parameter. The local effective shear rate was calculated from the strain-rate tensor as

$$\overset{\cdot }{\gamma }=\sqrt{2E_{\mathrm{sr}}:E_{\mathrm{sr}}},E_{\mathrm{sr}}=\frac{1}{2}\left[\nabla \vec{u}+{\left(\nabla \vec{u}\right)}^{T}\right]$$ (26)

This formulation allows blood viscosity to vary with the local shear rate and therefore captures the transient shear-thinning response induced by ultrasound. Compared with the constant-viscosity Newtonian model, it provides a more representative description of local viscous damping, shear-stress distribution, and the rheological environment adjacent to the vessel wall.

### Animal preparation

All animal studies and experimental procedures were approved by the Medical and Laboratory Animal Ethics Committee of the Beijing Institute of Technology. All mice were purchased from SPF (Beijing) Biotechnology Co., Ltd. (Beijing, China). A total of 42 male C57 mice (6 to 8 wk old) were used in this study. For the pressure-comparison experiment, 30 mice were randomly assigned to 6 groups according to stimulation mode (single-frequency or TI ultrasound) and reference pressure amplitude $P_{d}$ (0.3, 0.4, or 0.5 MPa), with 5 mice in each group. An additional 12 mice were used for the post-sonication permeability assessment and assigned to 4 post-sonication time points (0.5, 3, 6, and 12 h), with 3 mice at each time point. The mice were housed at 24 °C and 65 ± 15% relative humidity under a 12-h light/dark cycle. After 1 week of acclimatization, anesthesia was induced with 2% isoflurane in O_2_ (0.3 L/min) and maintained through a customized mask after the mice were placed in a stereotaxic apparatus. The scalp hair was removed with clippers, and body temperature was maintained at 37 °C using a heating pad. Five minutes before ultrasound stimulation, the isoflurane concentration was reduced to 0.5% (v/v).

### Ultrasound stimulation protocol

The experimental setup is shown schematically in Fig. 1A. The 1.0- and 0.8-MHz signals were generated using 2 function generators (AFG31000; Tektronix, Beaverton, OR, USA), combined into a dual-frequency electrical drive signal, and amplified by a radiofrequency power amplifier (2200L; E&I, Rochester, NY, USA). A 1-MHz FUS transducer (aperture 20 mm and focal depth 50 mm) was used to stimulate the mouse brain. A collimator filled with ultrasonic coupling agent was fixed between the transducer output and the mouse’s head (3D-printed and photo-cured) to facilitate ultrasound penetration into the brain.

The acoustic collimator was positioned over the left hemisphere using stereotactic coordinates spanning −5.25 to −3.25 mm relative to bregma and −2.25 to −0.25 mm lateral to the midline. The acoustic-pressure field of the ultrasound transducer was measured in degassed water using a needle hydrophone (NH0200; PA, Dorchester, UK). The −6-dB focal-zone dimensions were 3 mm laterally and 10.5 mm axially.

Stimulation parameters were as follows: single-frequency ultrasound, 1.0 MHz, 40 Hz pulse repetition frequency (PRF), 1% duty cycle (DC), and 1 s interstimulus interval (ISI); TI ultrasound, 1.0 + 0.8 MHz, 40 Hz PRF, 1% DC, and 1 s ISI. The total treatment duration was 2 min (Fig. 1B). Acoustic exposure was parameterized using the reference pressure amplitude $P_{d}$. For single-frequency excitation, the acoustic amplitude was $P_{d}$, whereas for TI excitation, the amplitudes of the 1.0- and 0.8-MHz components were each set to $\frac{P_{d}}{\surd 2}$. This normalization resulted in the same RMS pressure and time-averaged squared pressure for the 2 stimulation modes. Accordingly, the reported pressure levels of 0.3, 0.4, and 0.5 MPa refer to the reference pressure amplitude $P_{d}$, rather than the instantaneous peak pressure of the composite TI waveform. SonoVue MBs (Bracco Imaging S.p.A., Milan, Italy) were used after standard reconstitution according to the manufacturer’s instructions. The reconstituted suspension contains approximately 2 × 10^8^ MBs/mL, with a mean diameter of approximately 2.5 μm and a reported size range of 1 to 11 μm. Each mouse received 60 μL of the MB suspension by retro-orbital intravenous injection 1 min before ultrasound exposure.

For the pressure-comparison experiment, Evans blue was administered intravenously 0.5 h after ultrasound stimulation, and the brains were harvested after a 24-h circulation period to assess BBB permeability.

To characterize the time course of BBB recovery after low-pressure TI ultrasound, an independent cohort of 12 mice was exposed to 0.3 MPa TI sonication and assigned to 4 post-sonication assessment time points (0.5, 3, 6, and 12 h; *n* = 3 per time point). Evans blue extravasation was assessed at each time point to characterize the temporal evolution of BBB permeability.

### Transducer calibration and acoustic field characterization

The ultrasound transducer and hydrophone were immersed in degassed water. The acoustic-pressure distribution was characterized using a needle hydrophone (NH0200; PA), and a 2-dimensional pressure map was acquired with a computer-controlled 3-axis translation stage.

### Statistics

Data are expressed as mean ± standard error of the mean (SEM). Statistical analyses were performed using GraphPad Prism. For the pressure-comparison experiment, 2-way ANOVA was performed with stimulation mode and reference pressure amplitude $P_{d}$ as factors, including their interaction. Pairwise comparisons between single-frequency and TI groups at each pressure level were performed using Šídák’s multiple-comparisons test. A value of *P* <0.05 was considered statistically significant.

## Ethical Approval

The study was approved by the Medical and Laboratory Animal Ethics Committee of Beijing Institute of Technology (protocol No. 2025-015) and Personal License (1124052300022). The authors have adhered to the ARRIVE guidelines.

## Data Availability

All original contributions presented in this study are included in the article. Further inquiries can be directed to the corresponding authors.
